# Global Infection Rate of Rotavirus C during 1980–2022 and Analysis of Critical Factors in the Host Range Restriction of Virus VP4

**DOI:** 10.3390/v14122826

**Published:** 2022-12-19

**Authors:** Simiao Zhao, Xinshun Jin, Lingling Zang, Ziwei Liu, Xiaobo Wen, Xuhua Ran

**Affiliations:** 1 School of Animal Science and Technology, Hainan University, Haikou 570228, China; 2 Hainan Health Vocational College, Hainan University, Haikou 570228, China; 3 College of Animal Science & Veterinary Medicine, Heilongjiang Bayi Agricultural University, Daqing 163319, China

**Keywords:** rotavirus C, infection rate, capsid protein VP4, P genotype, host range restriction

## Abstract

Information on rotavirus C (RVC) infection is lacking, partly because the prevalence of RVC among humans and animals worldwide is undefined. Data on the characteristics of the P genotype among RVC strains are also required. We performed systematic searches on the infection rates of RVC since 1980 based on the literature and gene sequences of the PubMed and GenBank databases. A phylogenetic tree of VP4 genes was constructed to evaluate the distribution of the P genotype of RVC from various hosts. The specific mutation motifs in VP8* with P [2]/P [4]/P [5] specificity were analyzed to elucidate their roles in host range restriction. The rate of RVC infection in humans has fallen from 3% before 2009 to 1%, whereas in animals it has risen from 10% to 25%. The P genotype of RVC showed strict host species specificity, and current human RVC infections are exclusively caused by genotype P [2]. In the VP8* hemagglutinin domain of the P [4]/P [5] genotype of swine RVC, specific insertion or deletion were found relative to the human P [2] genotype, and these motifs are a possible critical factor for host range restriction. Our findings highlight the need for further epidemiological surveillance, preventive strategies, and elucidation of the factors involved in the specific host range restriction of RVC-circulating strains.

## 1. Introduction

Rotavirus is a leading cause of gastrointestinal diarrhea in both infants and newborn animals worldwide [1]. According to the Global Burden of Disease Study published in 2019, rotavirus still causes an estimated 235,331 deaths per year, which is 19.11% of all deaths due to diarrhea globally [2]. Although the burden of diarrheal disease in humans has declined following the introduction of vaccines, rotavirus infection remains the leading cause of diarrheal death in infants and children, especially in developing countries [3]. Additionally, rotavirus can infect domesticated and wild animals, resulting in the death of newborn animals [1]. Generally, vaccination with broad cross-neutralizing efficacy is an important measure to reduce severe rotavirus-associated gastroenteritis and mortality [4]. However, genetic diversity arising from various mutations and reassortment can diminish protective immune responses, raising concerns about the long-term implementation and efficacy of rotavirus vaccines.

As a representative member of the *Sedoreoviridae* family, rotaviruses were first identified in 1963 and first isolated from human infants in 1973 [5,6]. Rotavirus subtype classification is divided into 10+2 groups (RVA-RVJ and two novel hypothesis subtypes RVK/RVL recently identified in the common shrews (*Sorex araneus*) sample [7]) based on the inner capsid protein VP6, among which rotavirus A (RVA) is the most common and has been widely studied [8]. Since the rotavirus structural proteins VP4 and VP7 induce specific neutralizing antibodies and protective immunity in the host, a binary classification system for VP4 and VP7 has been established [9]. Thereinto, the VP4 defines the rotavirus P genotype, while the VP7 determines the G genotype of rotavirus. In addition, previous studies have shown that a single P genotype RVA vaccine provides poor heterologous protection against RVA in cattle infected with various P genotypes. [10]. In contrast, in humans, vaccines with different G genotypes offer higher immune protection [11]. The specific reasons for the differences between the P genotype and the G genotype in cross-immunity protection are still unclear and need further analysis. Recently, the Rotavirus Classification Working Group developed a complete genome-based system of rotavirus genotypes based on the classification of 11 dsRNA segments of all rotaviruses [12]. However, the applicability of this new classification method to RVA has not been reflected in rotavirus C (RVC) because most wild RVC strains are difficult to culture under laboratory conditions, and the sequencing data of their complete genome fragments are unavailable, which makes it difficult for RVC to establish a complete genetic system based on 11 dsRNA fragments [13].

Commercially available human rotavirus vaccines have been designed for RVA. Although RVC infection has been reported to cause severe gastroenteritis in humans and animals, leading to a severe social and economic burden worldwide [14,15], the diseases associated with RVC have been overlooked, compared with RVA, as have the genetic evolution and pathogenesis of RVC. Therefore, comprehensive information on the genetic and evolutionary diversity of RVC and host-specific infections will be of great significance for preventing the diseases associated with the various groups of rotavirus infections in humans and animals.

RVC infection was first confirmed in piglets with diarrhea in the United States in 1980 [16], then was later detected in Japan [17], Russia [18], Australia [19], and India [20] resulting in a severe economic burden on the global animal industry. Furthermore, RVC was initially identified as a causative pathogen of diarrheal disease in infants in 1982 [21], compared with the detection of the first RVA in humans in the 1970s. Current research into the distribution and infection rate of RVC is significantly lacking compared with similar research into RVA. RNA viruses present higher genetic and evolutionary diversity because of inferior RNA polymerase proofreading activity compared with DNA viruses [22]. Furthermore, genome rearrangement and reassortment for dsRNA viruses, including RVC, is attributable to the high frequency of genetic diversity and immune escape [23].

The rotavirus VP4 plays a vital role in cell entry and viral virulence [24,25]. Moreover, VP4 is a trypsin-sensitive protein, which can be digested into peptides VP8* and VP5* by trypsin [26]. VP8* contains the main antigenic site of the VP4 and is responsible for serum-specific neutralization [27]. The VP8* appears globular at the VP4 spike and contains a hemagglutinin domain associated with host carbohydrate recognition [28,29]. Recently, some rotavirus structural studies have shown that galectin-like VP8* of human RVA and RVC recognize various host cellular glycans in a genotype-dependent manner [30,31]. Thus, three-dimensional (3D) conformational changes involving VP8* may affect rotavirus invasion events in host cells. Therefore, understanding the viral infection rate and genotype-specific differences is essential for the targeted prevention of RVC infection.

In this study, we describe the infection rate of RVC and the distribution of the P genotype of RVC in different hosts based on available genome sequences. We also investigated the possible effect of specific differential amino acid motifs in the VP4 of the predominant RVC circulating between humans and swine on host range restriction. Our findings may contribute to understanding RVC-interspecies restrictions and facilitate the prevention and control of related diseases.

## 2. Materials and Methods

### 2.1. Systematic Review of Literature Retrieval Strategies and Selection Criteria

We searched the literature in the PubMed database using the following MeSH words: rotavirus/rotavirus C/rotavirus group C/C rotavirus, with a publication period dated up until June 2022. Duplicated and unrelated papers were excluded. Two independent researchers screened the literature according to the paper’s title and abstract and extracted essential information, including author, publication year, the affected countries or regions, number of patients, type of affected host, and infection rate (Table S1). The literature on rotavirus C included in this study is listed in Table S1. The inclusion criteria were as follows: all research objects had diarrhea caused by various types of RVC infection, the samples were diarrhea feces, specific detection methods were used to identify RVC, and the collection area for the feces sample was presented. In cases of disagreement during the inclusion process, additional researchers made independent judgments based on our inclusion criteria. The global infection rate of RVC was calculated in a cross-sectional study [32] using Review Manager 5.4.1 software (Review Manager (RevMan) [Computer program]. Version 5.4. The Cochrane Collaboration, 2020). The statistics for the infection rate were analyzed and illustrated using GraphPad Prism 9.0.0 software (GraphPad Software, San Diego, CA, USA, www.graphpad.com (accessed on 1 November 2022), 2020).

### 2.2. VP4 Gene Retrieval and Sequence Recombination Detection among RVC

RVC VP4 complete gene sequences (uploaded between 1980 and June 2022) were downloaded from the GenBank database. Those with synthetic duplicate sequences were excluded. The Clustal W sequence alignment module of MEGA-X software [33] was used to analyze the nucleotide sequences and those with significant motif length differences were removed. Then, the recombination events of the RVC sequences were evaluated using RDP 5.0 software based on seven methods (RDP/Chimera/GeneConv/Bootscan/Maxchi/Siscan/3 Seq).

### 2.3. Phylogenetic Tree Construction of VP4 Gene Sequences of RVC and Identification of VP4 Genotypes among Hosts

Given that gene recombination events would affect the accuracy of a phylogenetic tree, sequences with possible recombination were excluded. Among the viral information in the GenBank database that was not labeled with the P genotype, we conducted an online BLAST analysis to determine the P genotypes labeled with ‘!’ annotations (Table S2). VP4 phylogenetic and molecular evolutionary analyses were performed using MEGA-X software [33] with bootstrap values calculated from 1000 replicates. The maximum-likelihood phylogenetic algorithm was used for the construction of the tree. 

### 2.4. Amino Acid Sequence Alignment and Specific 3D Structure of RVC Capsid Protein VP4

RVC VP4 amino acid sequences were aligned using the Clustal W module in the MEGA-X software. Conserved domain information for the VP4 of RVC was obtained from the NCBI website. We used the Protein Data Bank (PDB) online website (https://www.rcsb.org/ accessed on 15 July 2022) to query published structures of infectious rotavirus particles (PDB: 4V7Q) [34] and the RVC VP8* 3D structure (PDB: 5ZHG), and RVC VP8* in complex with A-type HBGA trisaccharide (PDB ID: 5ZHO) [30]. UCSF Chimera X software [35] was used for the structural analysis of RV and VP8*. The VP8* 3D structure of the swine source P [4]/P [5] genotypes was predicted based on the SWISS-MODEL (https://swissmodel.expasy.org/ accessed on 5 August 2022).

### 2.5. Prediction of B-Cell Epitopes of RVC VP4

Conformational transition in the 3D structure of viral capsid proteins may cause an alteration in major epitopes and subsequently affect the efficiency of the host immune system. Specific insertion or deletion motifs in the amino acid sequence of VP8* will inevitably change the original conformation of the VP4. We used B-cell epitope prediction to determine the effect of insertion and deletion of VP4-specific motifs on host range restriction for the predominant circulating P genotypes of RVC. We used the epitope prediction module based on the Virus Pathogen Resources online database (https://www.viprbrc.org/ accessed on 1 August 2022) to predict the linear B-cell epitope of the VP8* of human RVC. Meanwhile, the 3D structure of VP8* and the predicted B-cell epitopes were visualized using UCSF Chimera X software [35], and the epitopes were color-coded with annotation in the 3D structure.

## 3. Results

### 3.1. Current Status of RVC Infections Worldwide

**Figure 1:**
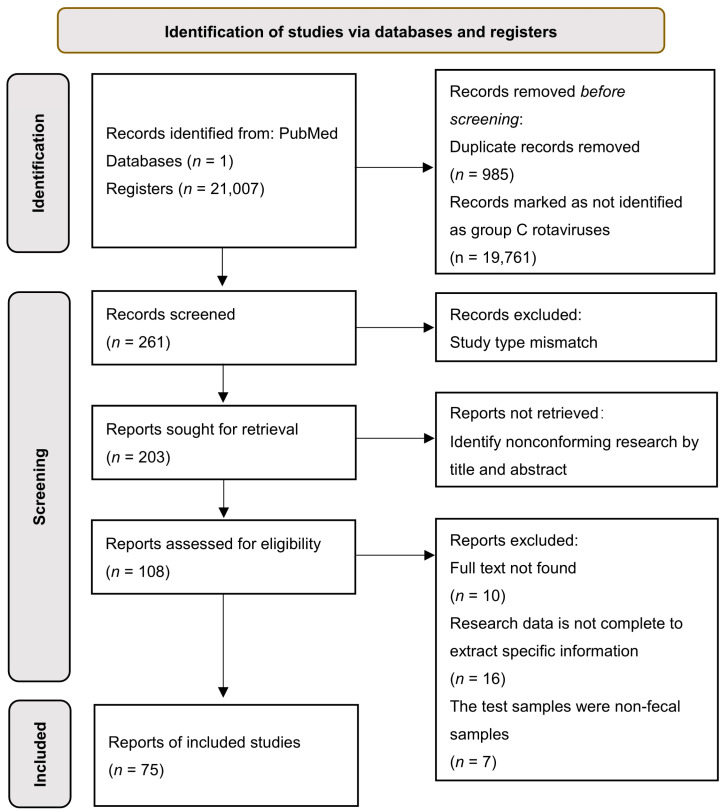
Flow diagram of the literature search and selection process for this study.

A total of 21,007 articles were retrieved in this study. Under the selection and screening strategy, 75 articles were included in this systematic analysis (Figure 1). Among the 75 reports on RVC infection, 49 were identified in humans, 22 in swine, 2 in bovine, 1 in canine, and 1 in mink. Regarding the geographical distribution of infection, 7 studies occurred in North America, 27 studies in Asia, 17 studies in South America, 2 studies in Africa, 21 studies in Europe, and 1 in Oceania.

**Figure 2:**
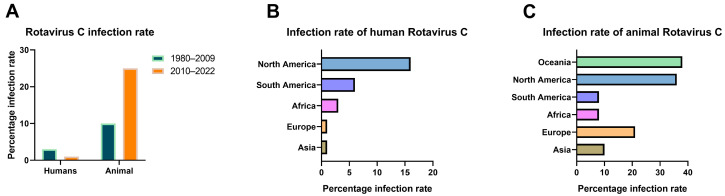
Bar chart of the RVC infection rates worldwide. (**A**) Bar chart of the RVC infection rates for 1980–2022, with the infected host as a partition unit and segmented nodes, as shown. (**B**) Bar chart of the infection rates of human infection with RVC at the intercontinental level. (**C**) Bar chart of the infection rates of RVC in animals (including swine, bovine, canine, and mink) across continents.

We calculated the infection rate of RVC based on data from the 75 studies (Supplementary data). Although there has been no report on the cross-protective effect of the RVA vaccine on RVC, considering that the World Health Organization included the RVA vaccine in the routine vaccination recommendations of all countries in 2009 [8], this measure may affect the detection of cases caused by RVC. We constructed a statistical bar chart of global RVC infections worldwide with 2009 as the time node (Figure 2A). The results showed that the average infection rate by human RVC was 3% during 1980–2009 and 1% during 2010–2022. This indicated an overall decline in infections of two percentage points and confirmed a downward trend in the human infection rate. In the case of animal rotavirus infection, the average infection rate was 10% during 1980–2009 and 25% during 2010–2022, revealing that the overall infection rate more than doubled during this time. We found that diarrhea samples (Figure 2B) showed the highest overall positive infection rate of 16% in humans in North America, with the lowest rate being in Europe and Asia at 1%. The infection rate of RVC in animals showed a regular distribution (Figure 2C). The overall infection rate in some economically and medically advanced nations, such as North America, Europe, and Oceania, was generally higher than in South America, Asia, and Africa. The highest infection rate was 38% in Oceania, followed by 36% in North America, 21% in Europe, 10% in Asia, 8% in South America, and 8% in Africa. Overall, the data indicated a complex profile of RVC infection. The host range for RVC infection is comprehensive, and the infection rate varies considerably among different countries and regions, with the infection spreading across all continents of the world.

### 3.2. Evolution of RVC VP4 and the Distribution of P Genotypes

**Figure 3:**
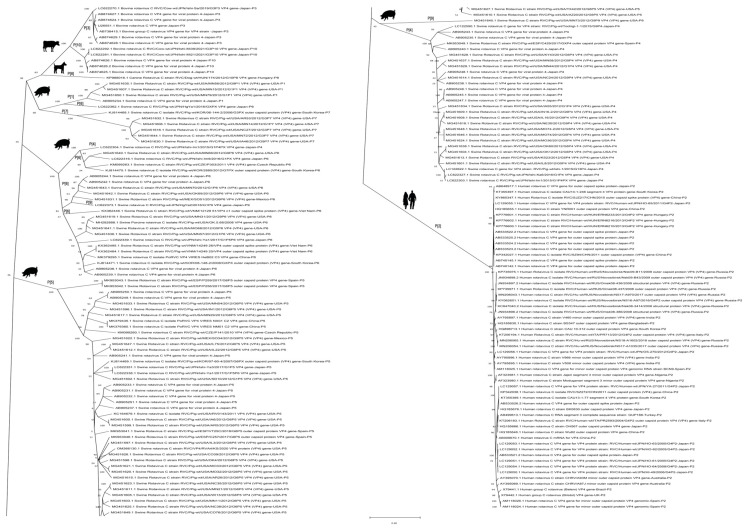
Phylogenetic tree of RVC VP4. The phylogenetic tree was constructed using the maximum-likelihood method based on the GTR+G+I model, and bootstrap values were calculated with 1000 replicates. The sequence information used in the phylogenetic analysis is shown in Table S2.

The results of recombination detection indicated that 16 sequences had undergone possible recombination (Table S3), with a recombination probability of 8.2%. Note that all of the RVC strains with possible recombination events on the rotavirus VP4 gene were isolated from swine, and no cross-species gene recombination was discovered. After that, a total of 175 VP4 sequences of RVC were used to construct a phylogenetic tree (Figure 3). The results showed that RVC is currently clustered into six branches in the VP4 phylogenetic tree. Meanwhile, a phylogenetic analysis of the RVC VP4 gene showed that the host source of the current RVC strains in the six clade branches had strict interspecies constraints. Therefore, we hypothesize that there is some unknown limiting factor on RVC VP4 that constrains the applicability of VP4 gene rearrangements in different host species.

To assess the distribution of the P genotype of RVC in various hosts, we highlighted the P genotype of each strain in the phylogenetic tree construction (Figure 3). Notably, we found that human RVC currently possesses only one P genotype, P [2] (Figure 3). In addition, RVC of genotype P [2] was exclusively isolated from humans, not animals. The genotypes of RVC detected in swine were more varied, including P [1]/P [4]/P [5]/P [6]/P [7]/P [9]. However, the P [4]/P [5]/P [6] rotaviruses were the predominant types, and the P [4]/P [5] genotype was mainly distributed in Asia, North America, and Europe. The P genotypes of RVC in bovine were exclusively P [3] and P [10], and their geographical distribution was limited to Japan. Canine RVC has only been documented for the Hungarian genotype P [8] strain.

### 3.3. Specificity Differences of RVC VP4 between Human P [2] and Swine P [4]/P [5]

**Figure 4:**
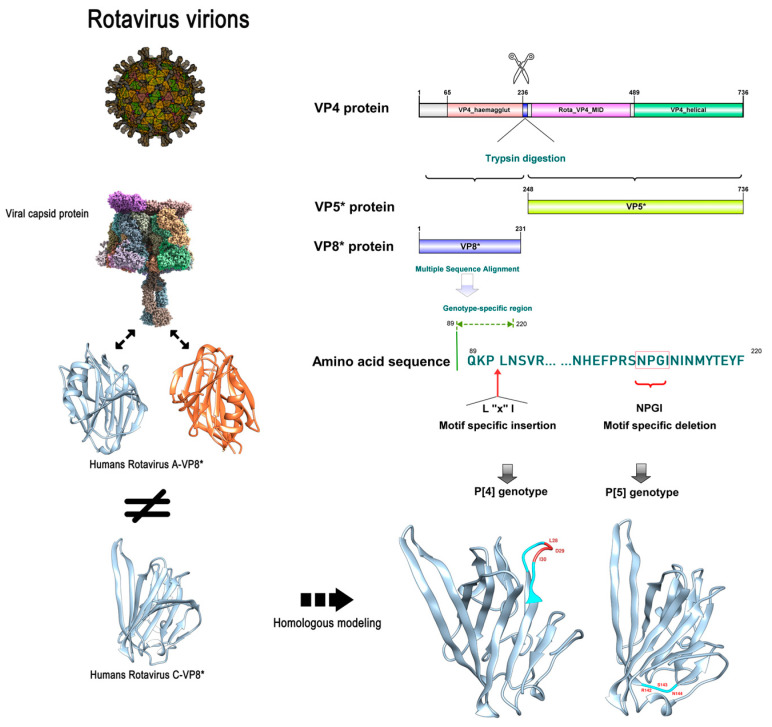
Schematic diagram of the specificity differences of the inserted and deleted motifs for RVC genotype P [2]/P [4]/P [5]. The structure of rotavirus virions and the VP8* structure of RVA were obtained from the Protein Data Bank online website (PDB: 4V7Q). RVC VP8* 3D structure (PDB: 5ZHG). The VP8* 3D structure of the P [4]/P [5] genotype was obtained by SWISS-MODEL homology modeling. In addition, the specific differences are as follows: for human P [2], swine P [4] has the insertion of the ‘L-X-I’ (where X is an unknown amino acid) motif, while swine P [5] has the deletion of the ‘N-P-G-I’ motif.

We randomly selected ten amino acid sequences from each VP4 amino acid sequence of RVC genotype P [2] and P [4]/P [5], respectively, and performed amino acid sequence alignment. Compared to the human P [2] genotype, we found that the VP4 gene with the P [4]/P [5] genotype had specific amino acid sequence mutations in specific regions (Figure 4). In the 90-amino acid VP4 of the P [4] genotype, a three-amino acid insertion motif was detected (‘L-X-I’, where X is an unknown amino acid). By contrast, RVC of genotype P [5] showed a four-amino acid deletion motif (‘N-P-G-I’) at amino acid 208 of VP4.

We found that the 3D structure of VP8* of human RVC differed from that of human RVA. In addition, the 3D structure showed that the specific insertion of swine rotavirus VP4 into the L-X-I motif of the P [4] genotype was located at the β-corner of the random coil, while the specific deletion motif of the P [5] genotype was also located in the other β-corner of the random coil (Figure 4).

### 3.4. Specific Differences in Swine P [4]/P [5] Genotypes Potentially Affecting Viral Invasion into Human Cells

**Figure 5:**
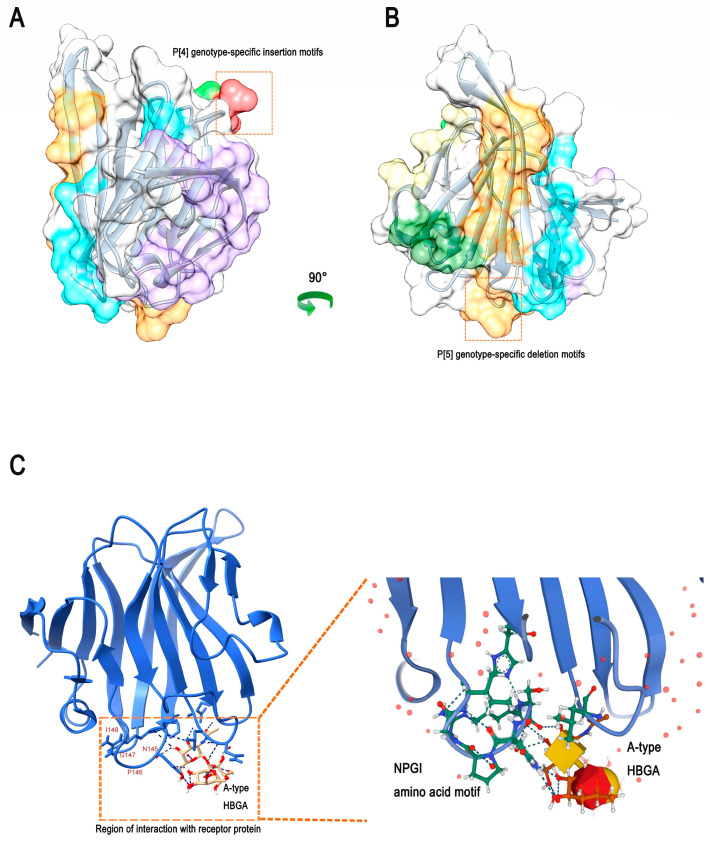
Prediction of human RVC VP8* B-cell epitopes and the recognition of host cell glycan complexes. (**A**) Shows a 90°-flip of the image in (**B**). A total of seven epitopes were predicted, the first of which was ‘I-E-W-S-N-L-I’, as highlighted in purple. The second was ‘L-N’, as highlighted in red. The third was ‘G-K-S-G-T-T’, as highlighted in light green. The fourth was ‘Q-N-K-T-H-D-A-N-S’, as highlighted in gold. The fifth was ‘E-G-S-T-Q-L’, as highlighted in dark green. The sixth was ‘V-G-G-I-L-I-K-P-I-N-S-S’, as highlighted in blue. The seventh was ‘N-W-N-H-E-F-P-R-S-N-P-G-I-N’, as highlighted in orange. The position of the P [4]/P [5] genotype-specific insertion or deletion motif of swine RVC is indicated by red boxes. (**C**) Human RVC recognition of the host cell glycan complex VP8* protein 3D structure (PDB: 5ZHO).

The epitope density of a pathogen is essential for inducing an effective host-specific immune response. In addition, given that the host response to virus-specific neutralizing antibodies is usually directed against the capsid protein of the virus itself, we find that the difference in the 3D structure of rotavirus VP8* between the P genotype-specific motifs lies in the random coil and the structural edges. Therefore, we speculate that these specific insertions or deletions of motifs may affect and alter the B-cell epitopes. We predicted the VP8* epitopes for RVC to explore whether specific insertion or deletion of the motif affects the host immune response to different P genotypes. In Figure 5A, the B-cell epitope (indicated in red) is one of the recognition epitopes of rotavirus, and the specific motif of P [4] rotavirus is inserted at this site. Another predicted B-cell epitope (indicated in orange) contains a P [5] genotype-specific deletion motif, which also inevitably alters the original epitope (Figure 5B). Furthermore, the process by which rotavirus attaches to glycosylated receptors on cell surfaces has been extensively demonstrated for host-specific and zoonotic potential effects. We found that the amino acid sequence of the ‘N-P-G-I’ motif with the specific deletion of swine P [5] genotypes was located at an important interaction site between human RVC and the receptor–glycan complex bound to host cells (Figure 5C). These results further indicate that the specific insertion or deletion of motifs in the P [4]/P [5] genotypes may be potential specific epitope sites affecting the host range restriction of RVC VP4.

## 4. Discussion

Despite the active implementation of commercial rotavirus vaccination worldwide, the diarrheal disease caused by rotavirus remains a threat to animal and public health. Disease induced by rotavirus infection is responsible for a significant economic burden, both on healthcare systems and the animal industry [36]. Furthermore, animal-original rotaviruses may be a source of human infection because of potential interspecies infection, resulting in the rise of genetic diversity and distinct serogroups. In this study, we focused on the infection rate of RVC, which has been neglected to date, and the specific differences in the predominant P genotypes. Specific insertion and deletion motifs were identified in the hemagglutinin domain of the VP8*. These motifs are presumed to be one of the key factors by which VP4 limits the host range of RVC invasion. 

Research into the diseases associated with RVC infection has been lacking, partly because of limited knowledge regarding viral prevalence in humans and animals worldwide. Our analysis demonstrated that the overall infection rate of RVC in humans is decreasing, although rates varied between different geographical regions. It is worth noting that before 2000, most of the detection methods for RVC were confined to ELISA or PAGE. With the development of the RT-PCR or real-time quantitative PCR technique, this more sensitive method of detection has been used. Thus, the true infection rate of RVC prior to 2009 may be higher than our statistical estimate [37]. However, we demonstrated that the RVC infection rate in animals has risen in recent years. In addition, infection rates for RVC are higher in economically and medically advanced intercontinental countries. For example, Europe and America showed a higher infection rate for RVC, whereas Asia, Africa, and South America showed a comparatively low infection rate. We think that these contradictory data reflect the positive correlation between economic status and medical facilities and the level of attention paid to animal infections [38]. Some developed countries that have focused on the detection and reporting of animal diseases have reported higher positive rates. Meanwhile, developed countries that have developed a higher degree of intensive breeding, a higher density of animal husbandry, and frequent trade of genetically-bred swine in varied feedlots have potentially promoted the cross-infection of RVC [39] and thereby exhibit a higher infection rate compared with developing countries.

The adaptive evolutionary process of viruses is crucial for their epidemic spread, immune evasion, and survival [40]. In our study, we found a high frequency of gene recombination in the VP4 gene of RVC, and all of the recombination events occurred exclusively in swine. Rotaviruses are assumed to possibly use swine as an intermediate carrier and undergo genome recombination to break the constraints of interspecies specificity. Some studies have revealed interspecies rearrangement in NSP4 and NSP5 of RVC [41]. However, our data based on a phylogenetic tree of RVC VP4 did not detect strains capable of cross-species transmission of VP4 among RVC-circulating strains. Therefore, we propose that rotavirus structural proteins VP4 play a more vital role in host range restriction than non-structural proteins NSP4 or NSP5.

Cross-species transmission is an evolutionary strategy for viral survival [42]. However, the majority of rotavirus strains exhibit a limited host range [43]. Recent findings on the cross-species transmission of rotaviruses challenge the consensus on the host range restriction of rotaviruses [44,45,46]. In this study, we found specific insertion or deletion motifs in the hemagglutinin domain of VP4 in the major P genotype of circulating RVC strains, which may be one of the important target factors of rotavirus VP4 for host range restriction [29]. Li and colleagues’ study on the specific binding of VP8* of rotavirus to host receptors found that the key binding region and the electrostatic effector region of VP8* of RVC were located around the ‘N-P-G-I’ motif at amino acid 208 [47]. The hypothesis is that the deletion of the amino acid motif ‘N-P-G-I’ will change the original VP8* conformation to bind to the host receptor and that virus invasion into the specific host will be affected. In other studies, the influence of the hemagglutinin domain on segmental virus virulence and cell invasion has been demonstrated [48,49,50]. However, the role of additional insertion or deletion mutations in the P [4]/P [5] VP4 on RVC entry and pathogenesis remains unknown. Therefore, further studies are needed to elucidate the function of a specific domain or modification of VP4 on RVC pathogenesis in both humans and animals. 

Our study had certain limitations. First, we did not consider gray literature in our search for reports on RVC infection. Gray literature includes data from unpublished papers, conference reports, or master/doctoral papers. In addition, in determining the distribution of RVC genotypes, we performed a BLAST comparison to calculate the genotype of strains not genotyped in the GenBank database; however, this may have led to the mislabeling of uncommon or previously undisclosed genotypes. Nonetheless, we believe that this did not affect our judgment of the distribution trend of RVC genotypes, and the homology between provisional P genotype strains confirmed by BLAST analysis in this study was higher than 85%.

In summary, this study broadens our understanding of the worldwide infection rate of RVC and the distribution of genotypes in host species. In addition, the specific differential characterization of VP4 of the major P genotype of RVC may play an important role in designing vaccine candidates, specific therapeutics, and immunodiagnostics for RVC.

## Data Availability

Not applicable.

## Supplementary Materials

The following supporting information can be downloaded at: https://www.mdpi.com/article/10.3390/v14122826/s1, Table S1: Rotavirus C infection statistics; Table S2: Phylogenetic tree strain information of rotavirus C; Table S3: Information of recombination events of the rotavirus C VP4; Supplemental data: The infection rate of Rotavirus C was analyzed by cross-sectional study data.
