# Supplementary material for: Global Infection Rate of Rotavirus C during 1980–2022 and Analysis of Critical Factors in the Host Range Restriction of Virus VP4
Source: Viruses. 2022 Dec 19;14(12):2826. doi: 10.3390/v14122826 (PMC9781963; doi:10.3390/v14122826)
Supplement: Supplementary file 1 [file viruses-14-02826-s001.zip › Supplementary Tables.zip!/Table S1.pdf]

Supplementary Table 1: Group C rotavirus infection statistics

Sequence number	Study ID	Sample Collection year	Sample Collection Site	Sample Collection Quantity	Group C rotavirus Positive cases	Group C rotavirus prevalence	Age of patients	Host species
1	Portal *et al.*, 2022	2017-2019	Brazil	93	1	1.08%	Adult > 65 years of age	Humans
2	Roczo-Farkas *et al.*, 2021	2020	Australia	111	42	37.84%	piglets	Swine
3	Costa *et al.*, 2020	2010-2016	Brazil	579	53	9.15%	unknown	Swine
4	Kim *et al.*, 2020	2016-2017	South Korea	801	0	0%	Babies and infants	Humans
5	Wu *et al.*, 2020	2015-2016	China	6051	10	0.17%	unknown	Humans
6	Campanha *et al.*, 2020	2019	Brazil	206	45	21.84%	piglets up to 7 days old	Swine
7	Cortey *et al.*, 2019	2017	Spain	47	19	40.43%	piglets up to 7 days old	Swine
8	Chepngeno *et al.*, 2019	Unknown	USA	113	86	76.10%	piglets	Swine
9	Vidal *et al.*, 2019	2017-2018	Spain	215	84	39.07%	piglets up to 7 days old	Swine
10	Xie *et al.*, 2018	2014-2017	Canada	527	14	2.66%	unknown	Mink
11	Tuanthap *et al.*, 2018	2011-2016	Thailand	769	51	6.63%	piglets	Swine
12	Bhat *et al.*, 2018	2010-2013	India	119	38	31.93%	Babies	Humans
13	Kowada *et al.*, 2018	2010-2013	Japan	382	3	0.79%	Babies	Humans
14	Kattoor *et al.*, 2017	Unknown	India	108	13	12.04%	piglets	Swine
15	Goecke *et al.*, 2017	Unknown	Denmark	989	5	0.51%	piglets	Swine
16	Amimo *et al.*, 2017	2012-2013	East Africa	446	37	8.30%	piglets aged 1–6 months	Swine
17	Tiku *et al.*, 2017	2013-2014	India	180	6	3.34%	Babies and infants	Humans
18	Mori *et al.*, 2017	1966-1983	Japan	307	3	0.98%	unknown	Humans
19	Joshi *et al.*, 2017	2006-2014	India	400	23	5.75%	adult	Humans
20	Zhirakovskaia *et al.*, 2016	2006-2011	Russia	2144	19	0.89%	adult and infants	Humans
21	Possatti *et al.*, 2016	2014	Brazil	1370	42	3.07%	unknown	Swine

22	Lobo Pdos *et al.*, 2016	2008-2011	Brazil	279	6	2.15%	Babies and infants	Humans
23	Theuns *et al.*, 2016	2014	Belgian	45	22	48.89%	piglets up to 14 days old	Swine
24	Zhou *et al.*, 2016	2010-2013	Austria, Germany, Hungary, Spain and Sweden	419	13	3.10%	all	Swine
25	Moutelíková *et al.*, 2015	2010-2013	Czech Republic	448	118	26.33%	all	Swine
26	Stipp *et al.*, 2015	2004-2010	Brazil	588	15	2.55%	unknown	Swine
27	Marthaler *et al.*, 2014	2009-2011	USA, Mexico, and Canada	7508	3833	51.05%	all	Swine
28	Moutelíková *et al.*, 2014	2009-2011	Czech Republic	293	75	25.60%	all	Swine
29	Kumazaki & Usuku, 2014	2006-2012	Japan	735	9	1.22%	unknown	Humans
30	Marthaler *et al.*, 2013	2009-2011	USA and Canada	7520	3447	45.84%	all	Swine
31	Ren *et al.*, 2013	2010	China	500	0	0%	children <5 years of age	Humans
32	Mori *et al.*, 2013	2008-2011	Japan,	8404	42	0.50%	unknown	Humans
33	Peng *et al.*, 2013	2007-2008	China	793	130	16.39%	all	Swine
34	Fukuda *et al.*, 2012	2002-2011	Japan	60	10	16.67%	adult cattle	Cattle
35	Lee *et al.*, 2011	2009	South Korea	166	2	1.20%	piglets	Swine
36	Luchs *et al.*, 2011	2007-2010	Brazil	3019	8	0.26%	children ≤15 years	Humans
37	Khamrin *et al.*, 2011	2008-2009	Japan	235	6	2.55%	Babies and infants	Humans
38	Moon *et al.*, 2011	2003-2006	South Korea	434	3	0.69%	children <10 years	Humans
39	Mitui *et al.*, 2009	2004-2005	Turkey	122	1	0.82%	children <5 years of age	Humans
40	Castello *et al.*, 2009	1997–2003	Argentina	1579	246	15.58%	children <17 years	Humans
41	Medici *et al.*, 2009	2004-2005	Italy	856	2	0.23%	children <10 years	Humans
42	Gabbay *et al.*, 2008	1982-1986	Brazil	77	5	6.49%	children <5 years of age	Humans
43	Collins *et al.*, 2008	2005-2007	Ireland	292	13	4.45%	4- to 5-week-old	Swine
44	Esona *et al.*, 2008	1999	USA	124	20	16.13%	children < or =10 years	Humans

45	Martella *et al.*, 2007	2007	Italy	188	54	28.72%	piglets	Swine
46	Nguyen *et al.*, 2007	2002-2003	Vietnam	1010	5	0.50%	Babies and infants	Humans
47	Abid *et al.*, 2007	1997-2000	Spain	467	3	0.64%	children <5 years of age	Humans
48	Bányai *et al.*, 2006	2003	Hungary.	472	3	0.63%	children ≤15 years	Humans
49	Logan *et al.*, 2006	2004-2005	Ireland	220	0	0%	Babies and infants	Humans
50	Gutierrez *et al.*, 2006	1999-2000	Colombia	300	57	19.00%	children <5 years of age	Humans
51	Phan *et al.*, 2006	2003-2004	Russia	100	1	1.00%	Babies and infants	Humans
52	Rahman *et al.*, 2005	2003	Bangladesh	611	14	2.29%	unknown	Humans
53	Phan *et al.*, 2005b	2002-2003	Japan	371	19	5.12%	Babies and infants	Humans
54	Phan *et al.*, 2005a	2002-2003	Japan	557	20	3.59%	Babies and infants	Humans
55	Phan *et al.*, 2004	2002-2003	Japan	236	24	10.17%	all	Humans
56	Kuzuya *et al.*, 2003	2000	Japan	32	21	65.63%	elementary school students	Humans
57	Sánchez-Fauquier *et al.*, 2003	2003	Spain	147	23	15.65%	Babies and infants	Humans
58	Castello *et al.*, 2002	1997-2000	Argentina	976	10	1.02%	ages 6 months to 15 years	Humans
59	Cunliffe *et al.*, 2001	1998-1999	Malawi	606	20	3.30%	children <5 years of age	Humans
60	Castello *et al.*, 2000	1996-1998	Argentina	500	90	18.00%	Babies and infants	Humans
61	Nilsson *et al.*, 2000b	1996-1997	Swedish	750	8	1.07%	>15 years old	Humans
62	Chang *et al.*, 1999	Unknown	USA	81	3	3.70%	adult cattle	Cattle
63	Gabbay *et al.*, 1999	1993	Brazil	64	8	12.5%	Babies and infants	Humans
64	Qiao *et al.*, 1999	1994-1996	China	186	1	0.54%	children (< or =5 years of age)	Humans
65	Otto *et al.*, 1999	1993-1997	Germany	23	3	13.04%	unknown	Canine
66	Teixeira *et al.*, 1998	1994	Brazil	406	5	1.23%	children <5 years of age	Humans
67	James *et al.*, 1998	1994-1995	UK	566	9	1.59%	all	Humans
68	Souza *et al.*, 1998	1993	Brazil	19	5	26.32%	all	Humans
69	Wu *et al.*, 1998	1994-1995	China	1385	1	0.07%	all	Humans

70	Jiang *et al.*, 1995	Unknown	USA	16	3	18.75%	Babies and infants	Humans
71	Maunula & von Bonsdorff, 1995	1986-1990	Finland	5316	8	0.15%	all	Humans
72	Rasool *et al.*, 1994	1987	Malaysia	334	1	0.30%	Babies and infants	Humans
73	Ludert & Liprandi, 1993	Unknown	Venezolano	214	1	0.47%	Babies and infants	Humans
74	Timenetsky Mdo *et al.*, 1993	1987-1988	Brazil	193	1	0.52%	Babies and infants	Humans
75	Sekine *et al.*, 1993	1991	Japan	11	6	54.55%	elementary school students	Humans
