# Supplementary material for: Global Infection Rate of Rotavirus C during 1980–2022 and Analysis of Critical Factors in the Host Range Restriction of Virus VP4
Source: Viruses. 2022 Dec 19;14(12):2826. doi: 10.3390/v14122826 (PMC9781963; doi:10.3390/v14122826)
Supplement: Supplementary file 1 [file viruses-14-02826-s001.zip › Supplementary Tables.zip!/Table S2.pdf]

Table S2: Phylogenetic tree strain information of group C rotaviruses

Host	GenBank access number	Collection Year	Country	P genotype	Name used for the phylogenetic tree
Bovine	AB874627	2004	Japan	P[3]!	AB874627.1 Bovine rotavirus C VP4 gene for viral protein 4-Japan-P3!
Bovine	AB874624	2003	Japan	P[3]!	AB874624.1 Bovine rotavirus C VP4 gene for viral protein 4-Japan-P3!
Bovine	U26551	1996	Japan	P[3]!	U26551.1 Bovine rotavirus C VP4 gene-Japan-P3!
Bovine	AB738415	2012	Japan	P[3]!	AB738415.1 Bovine group C rotavirus VP4 gene for VP4 strain -Japan-P3!
Bovine	AB874629	2010	Japan	P[3]!	AB874629.1 Bovine rotavirus C VP4 gene for viral protein 4-Japan-P3!
Bovine	AB874628	2008	Japan	P[3]!	AB874628.1 Bovine rotavirus C VP4 gene for viral protein 4-Japan-P3!
Bovine	LC622270	2019	Japan	P[3]	LC622270.1 Bovine rotavirus C RVC/Cow-wt/JPN/Ishi-Sa/2019/G3P3 VP4 gene-Japan-P3
Bovine	LC622292	2021	Japan	P[10]	LC622292.1 Bovine rotavirus C RVC/Cow-wt/JPN/Ishi-Mi39/2021/G3P10 VP4 gene-Japan-P10
Bovine	LC622281	2021	Japan	P[10]	LC622281.1 Bovine rotavirus C RVC/Cow-wt/JPN/Ishi-Mi21/2021/G3P10 VP4 gene-Japan-P10
Bovine	AB874626	2004	Japan	P[10]	AB874626.1 Bovine rotavirus C VP4 gene for viral protein 4-Japan-P10
Bovine	AB874626	2004	Japan	P[10]	AB874626.2 Bovine rotavirus C VP4 gene for viral protein 4-Japan-P10
Bovine	AB874625	2004	Japan	P[10]	AB874625.1 Bovine rotavirus C VP4 gene for viral protein 4-Japan-P10
Canine	KP988016	2012	Hungary	P[8]	KP988016.1 Canine Rotavirus C strain RVC/Dog-wt/HUN/174/2012/G10P8 VP4 gene-Hungary-P8
Swine	MG451635	2012	USA	P[1]	MG451635.1 Swine Rotavirus C strain RVC/Pig-wt/USA/MN56/2012/G9P1 VP4 (VP4) gene-USA-P1
Swine	MG451607	2012	USA	P[1]	MG451607.1 Swine Rotavirus C strain RVC/Pig-wt/USA/MN15/2012/G1P1 VP4 (VP4) gene-USA-P1
Swine	MG451650	2012	USA	P[1]	MG451650.1 Swine Rotavirus C strain RVC/Pig-wt/USA/MN79/2012/G1P1 VP4 (VP4) gene-USA-P1
Swine	AB905234	2002	Japan	P[1]!	AB905234.1 Swine rotavirus C VP4 gene for viral protein 4-Japan-P1!
Swine	LC622362	2016	Japan	P[6]!	LC622362.1 Swine rotavirus C RVC/Pig-wt/JPN/HgYa1/2016/GXPX VP4 gene-Japan-P6!
Swine	KJ814468	2006	South Korea	P[7]!	KJ814468.1 Swine rotavirus C isolate RVC/Pig-wt/KOR/06-144-2/2006/G3PX outer capsid protein (VP4) gene-South Korea-P7!
Swine	MG451632	2012	USA	P[7]	MG451632.1 Swine Rotavirus C strain RVC/Pig-wt/USA/AR53/2012/G3P7 VP4 (VP4) gene-USA-P7

Swine	MG451606	2012	USA	P[7]	MG451606.1 Swine Rotavirus C strain RVC/Pig-wt/USA/MN14/2012/G1P7 VP4 (VP4) gene-USA-P7
Swine	MG451616	2012	USA	P[7]	MG451616.1 Swine Rotavirus C strain RVC/Pig-wt/USA/NC27/2012/G3P7 VP4 (VP4) gene-USA-P7
Swine	MG451644	2012	USA	P[7]	MG451644.1 Swine Rotavirus C strain RVC/Pig-wt/USA/MN72/2012/G3P7 VP4 (VP4) gene-USA-P7
Swine	MG451630	2012	USA	P[7]	MG451630.1 Swine Rotavirus C strain RVC/Pig-wt/USA/IA46/2012/G9P7 VP4 (VP4) gene-USA-P7
Swine	MG451640	2012	USA	P[6]	MG451640.1 Swine Rotavirus C strain RVC/Pig-wt/USA/MN66/2012/G9P6 VP4 (VP4) gene-USA-P6
Swine	LC622316	2016	Japan	P[6]!	LC622316.1 Swine rotavirus C RVC/Pig-wt/JPN/Ishi-Im9/2016/G1PX VP4 gene-Japan-P6!
Swine	KM099263	2011	Czech Republic	P[6]!	KM099263.1 Swine rotavirus C strain RVC/Pig-wt/CZE/P303/2011 VP4 (VP4) gene-Czech Republic-P6!
Swine	KJ814475	2012	South Korea	P[6]!	KJ814475.1 Swine rotavirus C isolate RVC/Pig-wt/KOR/2885/2012/G7PX outer capsid protein (VP4) gene-South Korea-P6!
Swine	LC622304	2015	Japan	P[4]	LC622304.1 Swine rotavirus C RVC/Pig-wt/JPN/Ishi-Im1/2015/G1P4PX VP4 gene-Japan-P4
Swine	AB905244	2008	Japan	P[6]!	AB905244.1 Swine rotavirus C VP4 gene for viral protein 4-Japan-P6!
Swine	AB905242	2008	Japan	P[6]!	AB905242.1 Swine rotavirus C VP4 gene for viral protein 4-Japan-P6!
Swine	MG451643	2012	USA	P[6]	MG451643.1 Swine Rotavirus C strain RVC/Pig-wt/USA/MN70/2012/G1P6 VP4 (VP4) gene-USA-P6
Swine	MG451642	2012	USA	P[6]	MG451642.1 Swine Rotavirus C strain RVC/Pig-wt/USA/OK69/2012/G9P6 VP4 (VP4) gene-USA-P6
Swine	MG451631	2012	Mexico	P[6]	MG451631.1 Swine Rotavirus C strain RVC/Pig-wt/MEX/SO51/2012/G6P6 VP4 (VP4) gene-Mexico-P6
Swine	LC622373	2016	Japan	P[9]	LC622373.1 Swine rotavirus C RVC/Pig-wt/JPN/HgYa2/2016/G1P9 VP4 gene-Japan-P9
Swine	KX362446	2012	Viet Nam	P[6]!	KX362446.1 Swine Rotavirus C strain RVC/Pig-wt/VNM/12129 51/VP4 c1 outer capsid spike protein (VP4) gene-Viet Nam-P6!
Swine	MG451619	2012	USA	P[6]	MG451619.1 Swine Rotavirus C strain RVC/Pig-wt/USA/MN31/2012/G9P6 VP4 (VP4) gene-USA-P6
Swine	MH282888	2008	USA	P[6]!	MH282888.1 Swine Porcine rotavirus C isolate RVC/Pig-wt/USA/OK.5.68/2008 VP4 gene-USA-P6!
Swine	MG451641	2012	USA	P[6]	MG451641.1 Swine Rotavirus C strain RVC/Pig-wt/USA/MO68/2012/G5P6 VP4 (VP4) gene-USA-P6
Swine	MG451636	2012	USA	P[6]	MG451636.1 Swine Rotavirus C strain RVC/Pig-wt/USA/MN57/2012/G1P6 VP4 (VP4) gene-USA-P6
Swine	LC622339	2017	Japan	P[6]!	LC622339.1 Swine rotavirus C RVC/Pig-wt/JPN/Ishi-Ya1/2017/G1P5P6 VP4 gene-Japan-P6!
Swine	KX362495	2012	Viet Nam	P[6]!	KX362495.1 Swine Rotavirus C strain RVC/Pig-wt/VNM/14249 26/VP4 outer capsid spike protein (VP4) gene-Viet Nam-P6!
Swine	KX362484	2012	Viet Nam	P[6]!	KX362484.1 Swine Rotavirus C strain RVC/Pig-wt/VNM/14249 25/VP4 outer capsid spike protein (VP4) gene-Viet Nam-P6!
Swine	MK379295	2017	China	P[6]!	MK379295.1 Swine rotavirus C isolate PoRVC VP4 VIRES HeB02 C3 VP4 gene-China-P6!
Swine	KJ814471	2008	South Korea	P[6]!	KJ814471.1 Swine rotavirus C isolate RVC/Pig-wt/KOR/08-148-2/2008/GXPX outer capsid protein (VP4) gene-South Korea-P6!

Swine	AB905236	2002	Japan	P[6]!	AB905236.1 Swine rotavirus C VP4 gene for viral protein 4-Japan-P6!
Swine	AB905239	2003	Japan	P[6]!	AB905239.1 Swine rotavirus C VP4 gene for viral protein 4-Japan-P6!
Swine	MK953043	2017	Spain	P[5]	MK953043.1 Swine Rotavirus C strain RVC/Pig-wt/ESP/P259/2017/G6P5 outer capsid protein VP4 gene-Spain-P5
Swine	MK953042	2017	Spain	P[5]	MK953042.1 Swine Rotavirus C strain RVC/Pig-wt/ESP/P256/2017/G6P5 outer capsid protein VP4 gene-Spain-P5
Swine	AB905250	2009	Japan	P[5]!	AB905250.1 Swine rotavirus C VP4 gene for viral protein 4-Japan-P5!
Swine	AB905249	2008	Japan	P[5]!	AB905249.1 Swine rotavirus C VP4 gene for viral protein 4-Japan-P5!
Swine	MG451633	2012	USA	P[5]	MG451633.1 Swine Rotavirus C strain RVC/Pig-wt/USA/MN54/2012/G6P5 VP4 (VP4) gene-USA-P5
Swine	MG451596	2012	USA	P[5]	MG451596.1 Swine Rotavirus C strain RVC/Pig-wt/USA/IN1/2012/G6P5 VP4 (VP4) gene-USA-P5
Swine	MG451617	2012	USA	P[5]	MG451617.1 Swine Rotavirus C strain RVC/Pig-wt/USA/MN29/2012/G6P5 VP4 (VP4) gene-USA-P5
Swine	MK379435	2017	China	P[5]!	MK379435.1 Swine rotavirus C isolate PoRVC VP4 VIRES NX01 C2 VP4 gene-China-P5!
Swine	MK379360	2017	China	P[5]!	MK379360.1 Swine rotavirus C isolate PoRVC VP4 VIRES NM01 C2 VP4 gene-China-P5!
Swine	KM099260	2010	Czech Republic	P[5]!	KM099260.1 Swine rotavirus C strain RVC/Pig-wt/CZE/P141/2010 VP4 (VP4) gene-Czech Republic-P5!
Swine	MG451622	2012	Mexico	P[5]	MG451622.1 Swine Rotavirus C strain RVC/Pig-wt/MEX/SO34/2012/G9P5 VP4 (VP4) gene-Mexico-P5
Swine	MG451647	2012	USA	P[5]	MG451647.1 Swine Rotavirus C strain RVC/Pig-wt/USA/IL75/2012/G6P5 VP4 (VP4) gene-USA-P5
Swine	MG451612	2012	USA	P[5]	MG451612.1 Swine Rotavirus C strain RVC/Pig-wt/USA/IL22/2012/G6P5 VP4 (VP4) gene-USA-P5
Swine	AB905241	2008	Japan	P[5]!	AB905241.1 Swine rotavirus C VP4 gene for viral protein 4-Japan-P5!
Swine	KJ814469	2007	South Korea	P[5]!	KJ814469.1 Swine rotavirus C isolate RVC/Pig-wt/KOR/07-60-4/2007/G6PX outer capsid protein (VP4) gene-South Korea-P5!
Swine	LC622351	2017	Japan	P[5]	LC622351.1 Swine rotavirus C RVC/Pig-wt/JPN/Ishi-Ya3/2017/G1P5 VP4 gene-Japan-P5
Swine	LC622338	2017	Japan	P[5]	LC622338.1 Swine rotavirus C RVC/Pig-wt/JPN/Ishi-Ya1/2017/G1P5P6 VP4 gene-Japan-P5
Swine	MG451602	2012	USA	P[5]	MG451602.1 Swine Rotavirus C strain RVC/Pig-wt/USA/SD10/2012/G1P5 VP4 (VP4) gene-USA-P5
Swine	AB905233	2002	Japan	P[5]!	AB905233.1 Swine rotavirus C VP4 gene for viral protein 4-Japan-P5!
Swine	AB905231	2002	Japan	P[5]!	AB905231.1 Swine rotavirus C VP4 gene for viral protein 4-Japan-P5!
Swine	AB905232	2002	Japan	P[5]!	AB905232.1 Swine rotavirus C VP4 gene for viral protein 4-Japan-P5!
Swine	AB905251	2010	Japan	P[5]!	AB905251.1 Swine rotavirus C VP4 gene for viral protein 4-Japan-P5!
Swine	AB905237	2002	Japan	P[5]!	AB905237.1 Swine rotavirus C VP4 gene for viral protein 4-Japan-P5!

Swine	KC164679	2011	USA	P[5]!	KC164679.1 Swine rotavirus C isolate RVC/Pig-wt/USA/RV0143/2011 VP4 (VP4) gene-USA-P5!
Swine	MG451600	2012	USA	P[5]	MG451600.1 Swine Rotavirus C strain RVC/Pig-wt/USA/IA6/2012/G6P5 VP4 (VP4) gene-USA-P5
Swine	MG451599	2012	USA	P[5]	MG451599.1 Swine Rotavirus C strain RVC/Pig-wt/USA/AR5/2012/G6P5 VP4 (VP4) gene-USA-P5
Swine	MK953041	2018	Spain	P[5]	MK953041.1 Swine Rotavirus C strain RVC/Pig-wt/ESP/VT25C/2018/G6P5 outer capsid protein VP4 gene-Spain-P5
Swine	MG451623	2012	USA	P[5]	MG451623.1 Swine Rotavirus C strain RVC/Pig-wt/USA/NC35/2012/G6P5 VP4 (VP4) gene-USA-P5
Swine	MG451611	2012	USA	P[5]	MG451611.1 Swine Rotavirus C strain RVC/Pig-wt/USA/MN21/2012/G6P5 VP4 (VP4) gene-USA-P5
Swine	MG451605	2012	USA	P[5]	MG451605.1 Swine Rotavirus C strain RVC/Pig-wt/USA/WI13/2012/G6P5 VP4 (VP4) gene-USA-P5
Swine	MG451625	2012	USA	P[5]	MG451625.1 Swine Rotavirus C strain RVC/Pig-wt/USA/NC38/2012/G6P5 VP4 (VP4) gene-USA-P5
Swine	MG451603	2012	USA	P[5]	MG451603.1 Swine Rotavirus C strain RVC/Pig-wt/USA/MN11/2012/G6P5 VP4 (VP4) gene-USA-P5
Swine	MG451649	2012	USA	P[5]	MG451649.1 Swine Rotavirus C strain RVC/Pig-wt/USA/CO76/2012/G6P5 VP4 (VP4) gene-USA-P5
Swine	MG451627	2012	USA	P[5]	MG451627.1 Swine Rotavirus C strain RVC/Pig-wt/USA/TX42/2012/G6P5 VP4 (VP4) gene-USA-P5
Swine	MG451610	2012	USA	P[5]	MG451610.1 Swine Rotavirus C strain RVC/Pig-wt/USA/AZ20/2012/G6P5 VP4 (VP4) gene-USA-P5
Swine	MK953046	2017	Spain	P[5]	MK953046.1 Swine Rotavirus C strain RVC/Pig-wt/ESP/C257/2017/G6P5 outer capsid protein VP4 gene-Spain-P5
Swine	MG451615	2012	USA	P[5]	MG451615.1 Swine Rotavirus C strain RVC/Pig-wt/USA/AR26/2012/G6P5 VP4 (VP4) gene-USA-P5
Swine	MG451597	2012	USA	P[5]	MG451597.1 Swine Rotavirus C strain RVC/Pig-wt/USA/IL3/2012/G6P5 VP4 (VP4) gene-USA-P5
Swine	OM366130	2012	USA	P[5]!	OM366130.1 Swine rotavirus C strain RVC/VP4/RVA4/KS/2020 VP4 protein (VP4) gene-USA-P5!
Swine	MG451626	2012	USA	P[5]	MG451626.1 Swine Rotavirus C strain RVC/Pig-wt/USA/CO39/2012/G6P5 VP4 (VP4) gene-USA-P5
Swine	MG451598	2012	USA	P[5]	MG451598.1 Swine Rotavirus C strain RVC/Pig-wt/USA/OK4/2012/G6P5 VP4 (VP4) gene-USA-P5
Swine	MG451621	2012	USA	P[5]	MG451621.1 Swine Rotavirus C strain RVC/Pig-wt/USA/MO33/2012/G6P5 VP4 (VP4) gene-USA-P5
Swine	MG451620	2012	USA	P[5]	MG451620.1 Swine Rotavirus C strain RVC/Pig-wt/USA/MO32/2012/G6P5 VP4 (VP4) gene-USA-P5
Swine	MG451645	2012	USA	P[9]	MG451645.1 Swine Rotavirus C strain RVC/Pig-wt/USA/MN73/2012/G9P9 VP4 (VP4) gene-USA-P9
Swine	LC122590	2015	Japan	P[4]	LC122590.1 Swine rotavirus C gene for VP4 strain: RVC/Pig-wt/Tochigi-1-1/2015/G9P4-Japan-P4
Swine	AB905243	2008	Japan	P[4]!	AB905243.1 Swine rotavirus C VP4 gene for viral protein 4-Japan-P4!
Swine	AB905235	2002	Japan	P[4]!	AB905235.1 Swine rotavirus C VP4 gene for viral protein 4-Japan-P4!
Swine	MK953049	2017	Spain	P[4]	MK953049.1 Swine Rotavirus C strain RVC/Pig-wt/ESP/C429/2017/GXP4 outer capsid protein VP4 gene-Spain-P4

Swine	AB905240	2003	Japan	P[4]!	AB905240.1 Swine rotavirus C VP4 gene for viral protein 4-Japan-P4!
Swine	MG451628	2012	USA	P[4]	MG451628.1 Swine Rotavirus C strain RVC/Pig-wt/USA/KY43/2012/G6P4 VP4 (VP4) gene-USA-P4
Swine	MG451637	2012	USA	P[4]	MG451637.1 Swine Rotavirus C strain RVC/Pig-wt/USA/MN58/2012/G5P4 VP4 (VP4) gene-USA-P4
Swine	MG451629	2012	USA	P[4]	MG451629.1 Swine Rotavirus C strain RVC/Pig-wt/USA/MN44/2012/G1P4 VP4 (VP4) gene-USA-P4
Swine	AB905248	2008	Japan	P[4]!	AB905248.1 Swine rotavirus C VP4 gene for viral protein 4-Japan-P4!
Swine	MG451614	2012	USA	P[4]	MG451614.1 Swine Rotavirus C strain RVC/Pig-wt/USA/NC24/2012/G9P4 VP4 (VP4) gene-USA-P4
Swine	AB905238	2002	Japan	P[4]!	AB905238.1 Swine rotavirus C VP4 gene for viral protein 4-Japan-P4!
Swine	AB905246	2008	Japan	P[4]!	AB905246.1 Swine rotavirus C VP4 gene for viral protein 4-Japan-P4!
Swine	AB905245	2008	Japan	P[4]!	AB905245.1 Swine rotavirus C VP4 gene for viral protein 4-Japan-P4!
Swine	AB905247	2008	Japan	P[4]!	AB905247.1 Swine rotavirus C VP4 gene for viral protein 4-Japan-P4!
Swine	MG451634	2012	USA	P[4]	MG451634.1 Swine Rotavirus C strain RVC/Pig-wt/USA/IA55/2012/G1P4 VP4 (VP4) gene-USA-P4
Swine	MG451609	2012	USA	P[4]	MG451609.1 Swine Rotavirus C strain RVC/Pig-wt/USA/IN16-2/2012/G9P4 VP4 (VP4) gene-USA-P4
Swine	MG451608	2012	USA	P[4]	MG451608.1 Swine Rotavirus C strain RVC/Pig-wt/USA/IL16/2012/G9P4 VP4 (VP4) gene-USA-P4
Swine	MG451618	2012	USA	P[4]	MG451618.1 Swine Rotavirus C strain RVC/Pig-wt/USA/NE30/2012/G9P4 VP4 (VP4) gene-USA-P4
Swine	MG451648	2012	USA	P[4]	MG451648.1 Swine Rotavirus C strain RVC/Pig-wt/USA/MO74-2/2012/G5P4 VP4 (VP4) gene-USA-P4
Swine	MG451646	2012	USA	P[4]	MG451646.1 Swine Rotavirus C strain RVC/Pig-wt/USA/MO74/2012/G5P4 VP4 (VP4) gene-USA-P4
Swine	MG451624	2012	USA	P[4]	MG451624.1 Swine Rotavirus C strain RVC/Pig-wt/USA/MO36/2012/G5P4 VP4 (VP4) gene-USA-P4
Swine	MG451638	2012	USA	P[4]	MG451638.1 Swine Rotavirus C strain RVC/Pig-wt/USA/OK60/2012/G6P4 VP4 (VP4) gene-USA-P4
Swine	MG451604	2012	USA	P[4]	MG451604.1 Swine Rotavirus C strain RVC/Pig-wt/USA/OK12/2012/G6P4 VP4 (VP4) gene-USA-P4
Swine	MG451613	2012	USA	P[4]	MG451613.1 Swine Rotavirus C strain RVC/Pig-wt/USA/KS23/2012/G5P4 VP4 (VP4) gene-USA-P4
Swine	MG451601	2012	USA	P[4]	MG451601.1 Swine Rotavirus C strain RVC/Pig-wt/USA/IL8/2012/G5P4 VP4 (VP4) gene-USA-P4
Swine	LC122622	2015	Japan	P[4]	LC122622.1 Swine rotavirus C gene for VP4 strain: RVC/Pig-wt/Ishi-1/2015/G13P4-Japan-P4
Swine	LC622327	2016	Japan	P[4]	LC622327.1 Swine rotavirus C RVC/Pig-wt/JPN/Ishi-Ka6/2016/G1P4 VP4 gene-Japan-P4
Swine	LC622303	2015	Japan	P[4]	LC622303.1 Swine rotavirus C RVC/Pig-wt/JPN/Ishi-Im1/2015/G1P4PX VP4 gene-Japan-P4
Human	AB648917	2011	Japan	P[2]!	AB648917.1 Human rotavirus C VP4 gene for outer capsid spike protein-Japan-P2!

Human	KT355397	2014	South Korea	P[2]	KT355397.1 Human rotavirus C isolate CAU14-1-246 segment 4 VP4 protein gene-South Korea-P2
Human	KY865347	2015	China	P[2]!	KY865347.1 Human Rotavirus C isolate RVC/SJZ217/CHN/2015 outer capsid spike protein (VP4) gene-China-P2!
Human	LC129055	2011	Japan	P[2]	LC129055.1 Human rotavirus C VP4 gene for VP4 protein strain: RVC/Human-wt/JPN/HO-65/2011/G4P2-Japan-P2
Human	HQ185655	2007	China	P[2]!	HQ185655.1 Human rotavirus C strain YNR001 outer capsid protein VP4 gene-China-P2!
Human	KP776601	2013	Hungary	P[2]	KP776601.1 Human rotavirus C strain RVC/Human-wt/HUN/ERN6233/2013/G4P2 VP4 gene-Hungary-P2
Human	KP776602	2013	Hungary	P[2]	KP776602.1 Human rotavirus C strain RVC/Human-wt/HUN/ERN6216/2013/G4P2 VP4 gene-Hungary-P2
Human	KP776600	2013	Hungary	P[2]	KP776600.1 Human rotavirus C strain RVC/Human-wt/HUN/ERN6210/2013/G4P2 VP4 gene-Hungary-P2
Human	AB533522	2008	Japan	P[2]!	AB533522.2 Human rotavirus C VP4 gene for outer capsid spike protein-Japan-P2!
Human	AB533525	2008	Japan	P[2]!	AB533525.2 Human rotavirus C VP4 gene for outer capsid spike protein-Japan-P2!
Human	AB533524	2008	Japan	P[2]!	AB533524.2 Human rotavirus C VP4 gene for outer capsid spike protein-Japan-P2!
Human	AB533523	2008	Japan	P[2]!	AB533523.2 Human rotavirus C VP4 gene for outer capsid spike protein-Japan-P2!
Human	KP342027	2011	China	P[2]!	KP342027.1 Human rotavirus C isolate RVC/SZ94/CHN/2011 outer capsid protein (VP4) gene-China-P2!
Human	AB740145	2012	Japan	P[2]!	AB740145.1 Human rotavirus C VP4 gene for outer capsid spike protein-Japan-P2!
Human	AB740142	2012	Japan	P[2]!	AB740142.1 Human rotavirus C VP4 gene for outer capsid spike protein-Japan-P2!
Human	KP735975	2009	Russia	P[2]!	KP735975.1 Human Rotavirus C isolate RVC/Human-wt/RUS/Novosibirsk/Nsk09-B11/2009 outer capsid protein VP4 (VP4) gene-Russia-P2!
Human	JN934898	2009	Russia	P[2]!	JN934898.2 Human rotavirus C isolate RVC/Human-wt/RUS/Novosibirsk/Nsk09-B43/2009 outer capsid protein VP4 (VP4) gene-Russia-P2!
Human	JN934897	2008	Russia	P[2]!	JN934897.2 Human rotavirus C isolate RVC/Human-wt/RUS/Omsk08-436/2008 structural protein VP4 (VP4) gene-Russia-P2!
Human	KP735971	2008	Russia	P[2]!	KP735971.1 Human Rotavirus C isolate RVC/Human-wt/RUS/Omsk08-437/2008 outer capsid protein VP4 (VP4) gene-Russia-P2!
Human	MN206043	2017	Russia	P[2]!	MN206043.1 Human rotavirus C strain RVC/Hu-wt/RUS/Novosibirsk/NS17-A970/2017 outer capsid protein VP4 (VP4) gene-Russia-P2!
Human	KY062651	2016	Russia	P[2]	KY062651.1 Human rotavirus C isolate RVC/Human-wt/RUS/Novosibirsk/NS16-A97/2016/G4P2 outer capsid protein VP4 (VP4) gene-Russia-P2

Human	KC847040	2008	Russia	P[2]!	KC847040.2 Human rotavirus C isolate RVC/Human-wt/RUS/Novosibirsk/Nsk08-3414/2008 structural protein VP4 (VP4) gene-Russia-P2!
Human	JN934896	2008	Russia	P[2]!	JN934896.2 Human rotavirus C isolate RVC/Human-wt/RUS/Omsk08-386/2008 structural protein VP4 (VP4) gene-Russia-P2!
Human	AY795897	2004	India	P[2]!	AY795897.1 Human rotavirus C strain V460 minor outer capsid protein VP4 (VP4) gene-India-P2!
Human	HQ185635	2005	Bangladesh	P[2]!	HQ185635.1 Human rotavirus C strain BS347 outer capsid protein VP4 gene-Bangladesh-P2!
Human	HQ896713	2010	South Korea	P[2]!	HQ896713.1 Human rotavirus C strain CAU 10-312 outer capsid protein VP4 gene-South Korea-P2!
Human	KT206194	2012	Italy	P[2]	KT206194.1 Human Rotavirus C strain RVC/Human-wt/ITA/PR713/2012/G4P2 outer capsid protein VP4 (VP4) gene-Italy-P2
Human	MN206065	2018	Russia	P[2]	MN206065.1 Human rotavirus C strain RVC/Hu-wt/RUS/Novosibirsk/NS18-A1602/2018 outer capsid protein VP4 (VP4) gene-Russia-P2
Human	MN206054	2017	Russia	P[2]	MN206054.1 Human rotavirus C strain RVC/Hu-wt/RUS/Novosibirsk/NS17-A1035/2017 outer capsid protein VP4 (VP4) gene-Russia-P2
Human	LC129056	2012	Japan	P[2]	LC129056.1 Human rotavirus C VP4 gene for VP4 protein strain: RVC/Human-wt/JPN/OS-270/2012/G4P2-Japan-P2
Human	AY795896	2004	India	P[2]!	AY795896.1 Human rotavirus C strain V966 minor outer capsid protein VP4 (VP4) gene-India-P2!
Human	AY795895	2004	India	P[2]!	AY795895.1 Human rotavirus C strain V508 minor outer capsid protein VP4 (VP4) gene-India-P2!
Human	AM118025	2005	Spain	P[2]!	AM118025.1 Human rotavirus C VP4 gene for minor outer capsid protein VP4 genomic RNA strain BCN9-Spain-P2!
Human	AF323981	2000	Nigeria	P[2]!	AF323981.1 Human rotavirus C strain Jajeri segment 3 minor outer capsid protein VP4 gene-Nigeria-P2!
Human	AF323980	2000	Nigeria	P[2]!	AF323980.1 Human rotavirus C strain Moduganari segment 3 minor outer capsid protein VP4 gene-Nigeria-P2!
Human	LC129057	2011	Japan	P[2]	LC129057.1 Human rotavirus C VP4 gene for VP4 protein strain: RVC/Human-wt/JPN/YA-27/2011/G4P2-Japan-P2
Human	KP342038	2011	China	P[2]!	KP342038.1 Human rotavirus C isolate RVC/SZ272/CHN/2011 outer capsid protein (VP4) gene-China-P2!
Human	KT355389	2013	South Korea	P[2]	KT355389.1 Human rotavirus C isolate CAU13-1-77 segment 4 VP4 protein gene-South Korea-P2
Human	AB533526	2009	Japan	P[2]!	AB533526.2 Human rotavirus C VP4 gene for outer capsid spike protein-Japan-P2!
Human	HQ185676	2008	Japan	P[2]!	HQ185676.1 Human rotavirus C strain BK0830 outer capsid protein VP4 gene-Japan-P2!
Human	AB499613	2005	Turkey	P[2]!	AB499613.1 Human rotavirus C RNA segment 3 complete sequence strain: GUP188-Turkey-P2!
Human	KT206183	2004	Italy	P[2]	KT206183.1 Human Rotavirus C strain RVC/Human-wt/ITA/PR2593/2004/G4P2 outer capsid protein VP4 (VP4) gene-Italy-P2
Human	HQ185666	2003	Japan	P[2]!	HQ185666.1 Human rotavirus C strain OH567 outer capsid protein VP4 gene-Japan-P2!

Human	HQ185646	2001	China	P[2]!	HQ185646.1 Human rotavirus C strain Wu82 outer capsid protein VP4 gene-China-P2!
Human	AB008670	1997	China	P[2]!	AB008670.1 Human rotavirus C mRNA for VP4-China-P2!
Human	LC129053	2005	Japan	P[2]	LC129053.1 Human rotavirus C VP4 gene for VP4 protein strain: RVC/Human-wt/JPN/HO-63/2005/G4P2-Japan-P2
Human	LC129052	2005	Japan	P[2]	LC129052.1 Human rotavirus C VP4 gene for VP4 protein strain: RVC/Human-wt/JPN/HO-62/2005/G4P2-Japan-P2
Human	AB533521	2006	Japan	P[2]!	AB533521.2 Human rotavirus C VP4 gene for outer capsid spike protein-Japan-P2!
Human	LC129051	2005	Japan	P[2]	LC129051.1 Human rotavirus C VP4 gene for VP4 protein strain: RVC/Human-wt/JPN/HO-61/2005/G4P2-Japan-P2
Human	LC129054	2008	Japan	P[2]	LC129054.1 Human rotavirus C VP4 gene for VP4 protein strain: RVC/Human-wt/JPN/HO-64/2008/G4P2-Japan-P2
Human	LC129050	2006	Japan	P[2]	LC129050.1 Human rotavirus C VP4 gene for VP4 protein strain: RVC/Human-wt/JPN/HI-49/2006/G4P2-Japan-P2
Human	AY395070	2003	Australia	P[2]!	AY395070.1 Human rotavirus C strain CHRV/A93M minor outer capsid protein VP4 gene-Australia-P2!
Human	AY395069	2003	Australia	P[2]!	AY395069.1 Human rotavirus C strain CHRV/A87J minor outer capsid protein VP4 gene-Australia-P2!
Human	X79441	1994	Brazil	P[2]!	X79441.1 Human group C rotavirus (Belem) VP4 gene-Brazil-P2!
Human	X79442	1994	UK	P[2]!	X79442.1 Human group C rotavirus (Bristol) VP4 gene-UK-P2!
Human	AM118026	2005	Spain	P[2]!	AM118026.1 Human rotavirus C VP4 gene for minor outer capsid protein VP4 genomic-Spain-P2!
Human	AM118024	2005	Spain	P[2]!	AM118024.1 Human rotavirus C VP4 gene for minor outer capsid protein VP4 genomic-Spain-P2!

Notice： Among the strains not labeled with P genotype in GenBank database, we conducted an online Blast analysis to determine P genotypes, which was labeled with '!' annotations
