# Supplementary material for: Global Infection Rate of Rotavirus C during 1980–2022 and Analysis of Critical Factors in the Host Range Restriction of Virus VP4
Source: Viruses. 2022 Dec 19;14(12):2826. doi: 10.3390/v14122826 (PMC9781963; doi:10.3390/v14122826)
Supplement: Supplementary file 1 [file viruses-14-02826-s001.zip › Supplementary Tables.zip!/Table S3.pdf]

Table S3: Information of recombination events of the Group C rotavirus VP4

Event number	Abbreviate virus strain	Major parent	Minor parent	Breakpoints (In alignment)		Average p-value of the detection methods						
				Beginning	Ending	RDP	GeneConv	BootScan	MaxChi	Chimera	SiScan	3Seq
1	MT181131（Swine）	MG451632（Swine）	KC164679（Swine）	1162	1882	3.532×10^{-61}	4.029×10^{-58}	1.657×10^{-50}	1.035×10^{-24}	7.753×10^{-26}	1.662×10^{-27}	6.146×10^{-90}
2	KJ814472（Swine）	KJ814469（Swine）	KJ814471（Swine）	1134	2244	1.274×10^{-40}	3.359×10^{-37}	2.212×10^{-35}	1.337×10^{-28}	1.247×10^{-30}	3.064×10^{-31}	1.503×10^{-88}
3	KJ814476（Swine）	MG451632（Swine）	KJ814475（Swine）	2241	1234	4.144×10^{-60}	1.840×10^{-50}	1.925×10^{-63}	2.643×10^{-28}	ND	7.946×10^{-48}	4.041×10^{-80}
4	KJ814470（Swine）	AB905236（Swine）	MG451608（Swine）	1218	2238	ND	8.004×10^{-26}	3.330×10^{-26}	9.460×10^{-22}	1.811×10^{-23}	3.435×10^{-28}	6.304×10^{-54}
5	KJ814474（Swine）	KJ814468（Swine）	KJ814471（Swine）	45	1231	2.960×10^{-18}	3.718×10^{-12}	1.991×10^{-16}	8.459×10^{-19}	1.477×10^{-8}	1.170×10^{-28}	9.695×10^{-42}
6	MK953047（Swine）	MK953040（Swine）	MK953046（Swine）	1860	2242	ND	2.105×10^{-37}	6.316×10^{-35}	4.135×10^{-16}	1.487×10^{-7}	8.755×10^{-17}	7.182×10^{-29}
7	KM099261（Swine）	MT181131（Swine）	KM099264（Swine）	864	1128	5.469×10^{-22}	3.303×10^{-31}	8.705×10^{-24}	3.600×10^{-12}	1.149×10^{-11}	4.191×10^{-15}	4.032×10^{-22}
8	KJ814473（Swine）	KJ814469（Swine）	KC164679（Swine）	2248	1234	2.619×10^{-7}	1.359×10^{-5}	7.696×10^{-4}	5.299×10^{-13}	5.782×10^{-5}	6.625×10^{-30}	7.553×10^{-29}
9	KM099264（Swine）	KJ814475（Swine）	AB905250（Swine）	1150	1594	3.228×10^{-16}	1.305×10^{-3}	1.517×10^{-11}	1.381×10^{-6}	1.537×10^{-2}	2.285×10^{-6}	1.949×10^{-6}
10	MK953045（Swine）	AB905233（Swine）	MK953041（Swine）	2238	658	1.578×10^{-10}	1.508×10^{-11}	ND	3.051×10^{-9}	4.902×10^{-7}	6.226×10^{-13}	ND
11	LC122611（Swine）	AB905235（Swine）	LC622373（Swine）	2244	147	ND	3.873×10^{-11}	3.189×10^{-7}	2.285×10^{-2}	2.194×10^{-2}	ND	3.483×10^{-8}
12	KM099262（Swine）	MG451632（Swine）	KJ814475（Swine）	872	1240	8.770×10^{-9}	ND	1.611×10^{-7}	3.399×10^{-4}	6.708×10^{-7}	2.194×10^{-3}	4.451×10^{-11}
13	MK953039（Swine）	MK953049（Swine）	MG451624（Swine）	514	1287	ND	ND	ND	5.245×10^{-8}	1.367×10^{-5}	3.363×10^{-7}	7.082×10^{-9}
14	MK953044（Swine）	MK953043（Swine）	MK953048（Swine）	254	898	2.107×10^{-7}	4.970×10^{-12}	1.283×10^{-11}	3.092×10^{-8}	3.472×10^{-5}	3.035×10^{-12}	2.567×10^{-3}
15	MK953048（Swine）	MG451621（Swine）	LC622339（Swine）	92	760	ND	ND	ND	3.286×10^{-5}	1.520×10^{-1}	5.470×10^{-3}	4.983×10^{-5}
16	MK953040（Swine）	MK953047（Swine）	KM099264（Swine）	628	760	ND	7.707×10^{-3}	ND	6.396×10^{-6}	1.063×10^{-3}	ND	2.621×10^{-5}

ND: Recombination not detected with this algorithm.

In alignment: The genome sequence has been aligned by the alignment correction module of the Mega-X software.

Abbreviate virus strain: The full name of each virus strain has been abbreviated in this study. The abbreviation is represented by the number of the virus strain in the GeneBank.
